# Supplementary material for: Quantum-enabled operation of a microwave-optical interface
Source: Nat Commun. 2022 Mar 11;13:1276. doi: 10.1038/s41467-022-28924-2 (PMC8917169; doi:10.1038/s41467-022-28924-2)
Supplement: Supplementary file 1 — Supplementary Information [file 41467_2022_28924_MOESM1_ESM.pdf]

Supplementary Information for: "Quantum-enabled operation of a microwave-optical interface"

Rishabh Sahu,[1,*] William Hease,[1] Alfredo Rueda,[1] Georg Arnold,[1] Liu Qiu,[1] and Johannes M. Fink[1,†]

[1] *Institute of Science and Technology Austria, am Campus 1, 3400 Klosterneuburg, Austria*

(Dated: February 7, 2022)

CONTENTS

Page

Supplementary Note 1. Theory 2

A Hamiltonian . . . 2

B Equations of motion . . . 2

C Coherent time-domain dynamics . . . 3

D Steady state model . . . 3

E Noise analysis . . . 4

Supplementary Note 2. Experimental Setup 5

Supplementary Note 3. System Characterization 5

A Low cooperativity characterization . . . 5

B Low cooperativity conversion . . . 8

C High cooperativity characterization . . . 9

Supplementary Note 4. 4-port calibration 10

Supplementary Note 5. Time dependence of transduction efficiency 10

Supplementary Note 6. Optical heterodyne detection 11

Supplementary Note 7. Microwave noise measurements 12

Supplementary Note 8. Kerr Effect 13

References 14

* rsahu@ist.ac.at

† jfink@ist.ac.at

Supplementary Note 1. THEORY

Our quantum transducer uses the $\chi^{(2)}$ nonlinearity in Lithium Niobate to directly couple microwave and optical fields [1, 2]. The nonlinearity is enhanced by utilizing a cavity resonance for all involved modes. A high quality whispering gallery mode resonator (WGMR) supports the optical modes. It is placed inside a circular microwave cavity whose $m = 1$ mode is in-situ frequency tunable and accurately matched with the optical FSR. We use the highest r_{33} electro-optic coefficient to couple the transverse electric (TE) polarized microwave and optical fields.

A. Hamiltonian

The microwave mode couples with the optical pump and the two optical sidebands on either side of the optical pump. The interaction Hamiltonian is given as,

$$\hat{H}_{\text{int}} = \hbar g_0(\hat{a}_e\hat{a}_p\hat{a}_o^\dagger + \hat{a}_e^\dagger\hat{a}_p\hat{a}_s^\dagger) + \text{h.c.}, \tag{1}$$

where g_0 is the non-linear vacuum coupling strength, and $\hat{a}_p$, and $\hat{a}_e$ represent optical pump mode and microwave mode annihilation operators respectively. $\hat{a}_o$ and $\hat{a}_s$ represent the optical sideband mode annihilation operators on the blue (optical signal mode) and the red (optical Stokes mode) side of the optical pump separated by one optical FSR. As noted in the main text, the first term in Eq. 1 represents a beam splitter interaction between the microwave mode $\hat{a}_e$ and the optical signal mode $\hat{a}_o$ as needed for noiseless conversion between microwave and optics fields. The second term is a two mode squeezing interaction due to interaction of the microwave mode $\hat{a}_e$ and the optical Stokes mode $\hat{a}_s$ also mediated by the optical pump mode. This term is responsible for phase insensitive amplification, which adds extra noise in the process of transduction and should be suppressed in an ideal transducer.

In our experiment, we suppress the participation of the optical mode $\hat{a}_s$ by coupling this mode to a degenerate transverse magnetic (TM) optical mode $\hat{a}_r$, as shown in Supplementary Fig. 1. This creates an avoided crossing in the TE mode $\hat{a}_s$ [3, 4]. The pump then interacts with this hybridized $\hat{a}_s$ mode reducing the density of states in the relevant frequency range. The full interaction Hamiltonian for our system is

$$\hat{H}_{\text{int}} = \hbar g_0(\hat{a}_e\hat{a}_p\hat{a}_o^\dagger + \hat{a}_e^\dagger\hat{a}_p\hat{a}_s^\dagger) + iJ\hat{a}_s^\dagger\hat{a}_r + \text{h.c.}, \tag{2}$$

where J is the coupling rate between the $\hat{a}_s$ and the $\hat{a}_r$ modes.

B. Equations of motion

We derive the time dynamics of these operators using the Heisenberg equations of motion. The resulting system of equations is the following:

$$\dot{\hat{a}}_p = -i\Delta_p\hat{a}_p - \tfrac{\kappa_o}{2}\hat{a}_p - ig_0(\hat{a}_s\hat{a}_e + \hat{a}_e^\dagger\hat{a}_o) + \Lambda\sqrt{\kappa_{o,\text{ex}}}\bar{F}_p + \sqrt{\kappa_{o,\text{ex}}}\delta\hat{a}_{p,e} + \sqrt{\kappa_{o,\text{in}}}\delta\hat{a}_{p,\text{in}}, \tag{3a}$$

$$\dot{\hat{a}}_o = -i\Delta_o\hat{a}_o - \tfrac{\kappa_o}{2}\hat{a}_o - ig_0\hat{a}_p\hat{a}_e + \Lambda\sqrt{\kappa_{o,\text{ex}}}\bar{F}_o + \sqrt{\kappa_{o,\text{ex}}}\delta\hat{a}_{o,\text{ex}} + \sqrt{\kappa_{o,\text{in}}}\delta\hat{a}_{o,\text{in}}, \tag{3b}$$

$$\dot{\hat{a}}_s = -i\Delta_s\hat{a}_s - \tfrac{\kappa_o}{2}\hat{a}_s - ig_0\hat{a}_p\hat{a}_e^\dagger - iJ\hat{a}_r + \sqrt{\kappa_{o,e}}\delta\hat{a}_{s,\text{ex}} + \sqrt{\kappa_{o,\text{in}}}\delta\hat{a}_{s,\text{in}}, \tag{3c}$$

$$\dot{\hat{a}}_e = -i\Delta_e\hat{a}_e - \tfrac{\kappa_e}{2}\hat{a}_e - ig_0\hat{a}_p\hat{a}_s^\dagger - ig_0\hat{a}_p^\dagger\hat{a}_o + \sqrt{\kappa_{e,\text{ex}}}\bar{F}_e + \sqrt{\kappa_{e,\text{ex}}}\delta\hat{a}_{e,\text{ex}} + \sqrt{\kappa_{e,\text{in}}}\delta\hat{a}_{e,\text{in}}, \tag{3d}$$

$$\dot{\hat{a}}_r = -i\Delta_r\hat{a}_r - \tfrac{\kappa_r}{2}\hat{a}_s - iJ\hat{a}_s + \sqrt{\kappa_r}\delta\hat{a}_{r,\text{in}}. \tag{3e}$$

Here, κ_j, $\kappa_{j,\text{ex}}$ and $\kappa_{j,\text{in}}$ are the total, extrinsic and intrinsic loss rates of respective modes, Δ_j are the detuning of the mode anhilation operators from their respective resonance frequencies, $\bar{F}_j$ are the coherent drive terms given by $|\bar{F}_j| = \sqrt{P_j/\hbar\omega_j}$ and $\delta\hat{a}_{j,\text{in}}$ and $\delta\hat{a}_{j,\text{ex}}$ represent the Langevin noise operators for bath and waveguide respectively. We take into account the finite mode matching between the free space coupled Gaussian mode of the single mode fiber and the evanescent field of the WGMR modes by multiplying $\kappa_{o,\text{ex}}$ with the mode amplitude overlap Λ [5]. The noise operators follow the following correlations

$$\langle\delta\hat{a}_{j,k}(t)\delta\hat{a}_{j,k}(t')^\dagger\rangle = (\bar{n}_k + 1)\delta(t - t'), \tag{4a}$$

$$\langle\delta\hat{a}_{j,k}(t)^\dagger\delta\hat{a}_{j,k}(t')\rangle = \bar{n}_k\delta(t - t'), \tag{4b}$$

where $k \in (\text{in}, \text{ex})$. For optics, both $\bar{n}_{\text{in}} = 0$ and $\bar{n}_{\text{ex}} = 0$, while for microwave, $\bar{n}_{\text{in}} = N_b$ and $\bar{n}_{\text{ex}} = N_{\text{wg}}$.

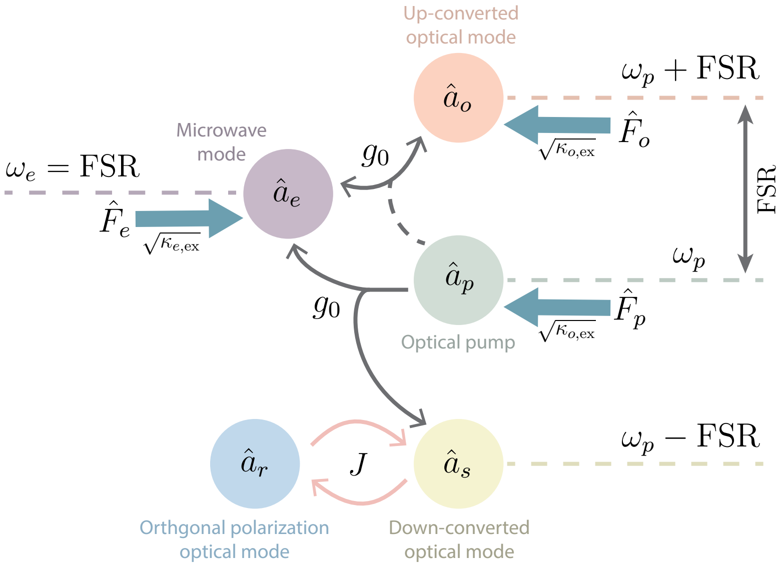

Supplementary Fig. 1. **Participating modes and their interactions.** In the presented converter, there are three optical TE modes - the optical pump mode $\hat{a}_p$ and one optical mode on the blue ($\hat{a}_o$) and red ($\hat{a}_s$) side of the optical pump separated by one FSR each. The optical mode $\hat{a}_o$ is parametrically coupled to the microwave mode $\hat{a}_e$ via the beam splitter interaction with the vacuum coupling rate g_0. The microwave mode $\hat{a}_e$ and the optical mode $\hat{a}_s$ also stimulate the optical pump $\hat{a}_p$ to down-convert into $\hat{a}_s$ and $\hat{a}_e$ via the two-mode squeezing interaction. This two-mode squeezing interaction is suppressed by coupling the optical mode $\hat{a}_s$ to a degenerate TM optical mode $\hat{a}_r$ with coupling strength J creating an anti-crossing at the $\hat{a}_s$ mode frequency. In our experiment, we use three coherent inputs - $\hat{F}_p$ for the optical pump, $\hat{F}_e$ for the microwave mode, and $\hat{F}_o$ for the optical signal mode to probe the system dynamics.

C. Coherent time-domain dynamics

We first focus on the coherent time domain dynamics in our quantum transducer. By linearizing the intra-cavity field for the modes $\hat{a}_j = \bar{a}_j + \delta\hat{a}_j$, with $\bar{a}_j$ being the coherent field amplitude and $\delta\hat{a}_j$ the field fluctuations, we obtain the following coherent dynamics for the optical and microwave modes:

$$\dot{\bar{a}}_p = -i\Delta_p\bar{a}_p - \tfrac{\kappa_o}{2}\bar{a}_p - ig_0(\bar{a}_s\bar{a}_e + \bar{a}_e^*\bar{a}_o) + \Lambda\sqrt{\kappa_{o,\mathrm{ex}}}\bar{F}_p, \tag{5a}$$
$$\dot{\bar{a}}_o = -i\Delta_o\bar{a}_o - \tfrac{\kappa_o}{2}\bar{a}_o - ig_0\bar{a}_p\bar{a}_e + \Lambda\sqrt{\kappa_{o,\mathrm{ex}}}\bar{F}_o, \tag{5b}$$
$$\dot{\bar{a}}_s = -i\Delta_s\bar{a}_s - \tfrac{\kappa_o}{2}\bar{a}_s - ig_0\bar{a}_p\bar{a}_e^* - iJ\bar{a}_e, \tag{5c}$$
$$\dot{\bar{a}}_e = -i\Delta_e\bar{a}_e - \tfrac{\kappa_e}{2}\bar{a}_e - ig_0\bar{a}_p\bar{a}_s^* - ig_0\bar{a}_p^*\bar{a}_o + \sqrt{\kappa_{e,\mathrm{ex}}}\bar{F}_e, \tag{5d}$$
$$\dot{\bar{a}}_r = -i\Delta_r\bar{a}_r - \tfrac{\kappa_r}{2}\bar{a}_r - iJ\bar{a}_s. \tag{5e}$$

The output fields are then calculated as $\bar{a}_{e,\mathrm{out}} = \sqrt{\kappa_{e,\mathrm{ex}}}\bar{a}_e - \bar{F}_e$ for the microwave mode and $\bar{a}_{o,\mathrm{out}} = \Lambda\sqrt{\kappa_{o,\mathrm{ex}}}\bar{a}_o - \bar{F}_o$ for the upconverted optics mode. The above system of equations are numerically solved in the time domain using Euler's method. We use this method to model the classical time-dynamics of the system with arbitrary coherent pump pulses, thereby, fitting the experimental results shown in Figs. 1 and 2 of main text.

D. Steady state model

After linearizing Eqs. 3, valid for a strong classical pump tone, we rewrite this equation set in matrix form as

$$\dot{\boldsymbol{v}}(t) = \boldsymbol{M}\boldsymbol{v}(t) + \boldsymbol{K}\boldsymbol{A}(t), \tag{6}$$

where $\boldsymbol{v}(t)$ is $[\delta\hat{a}_o, \delta\hat{a}_o^\dagger, \delta\hat{a}_e, \delta\hat{a}_e^\dagger, \delta\hat{a}_s, \delta\hat{a}_s^\dagger, \delta\hat{a}_r, \delta\hat{a}_r^\dagger]^T$,

$$\boldsymbol{M} = \begin{bmatrix} -i\Delta_o - \frac{\kappa_o}{2} & 0 & -ig & 0 & 0 & 0 & 0 & 0 \\ 0 & i\Delta_o - \frac{\kappa_o}{2} & 0 & ig^* & 0 & 0 & 0 & 0 \\ -ig^* & 0 & -i\Delta_e - \frac{\kappa_e}{2} & 0 & 0 & -ig & 0 & 0 \\ 0 & ig & 0 & i\Delta_e - \frac{\kappa_e}{2} & ig^* & 0 & 0 & 0 \\ 0 & 0 & 0 & -ig & -i\Delta_s - \frac{\kappa_s}{2} & 0 & -iJ & 0 \\ 0 & 0 & ig^* & 0 & 0 & i\Delta_s - \frac{\kappa_s}{2} & 0 & iJ \\ 0 & 0 & 0 & 0 & -iJ & 0 & -i\Delta_r - \frac{\kappa_r}{2} & 0 \\ 0 & 0 & 0 & 0 & 0 & iJ & 0 & i\Delta_r - \frac{\kappa_r}{2} \end{bmatrix}, \tag{7}$$

$$\boldsymbol{K} = \begin{bmatrix} \sqrt{\kappa_{o,\text{in}}} & 0 & \sqrt{\kappa_{o,\text{ex}}} & 0 & 0 & 0 & 0 & 0 & 0 & 0 & 0 & 0 & 0 & 0 \\ 0 & \sqrt{\kappa_{o,\text{in}}} & 0 & \sqrt{\kappa_{o,\text{ex}}} & 0 & 0 & 0 & 0 & 0 & 0 & 0 & 0 & 0 & 0 \\ 0 & 0 & 0 & 0 & \sqrt{\kappa_{e,\text{in}}} & 0 & \sqrt{\kappa_{e,\text{ex}}} & 0 & 0 & 0 & 0 & 0 & 0 & 0 \\ 0 & 0 & 0 & 0 & 0 & \sqrt{\kappa_{e,\text{in}}} & 0 & \sqrt{\kappa_{e,\text{ex}}} & 0 & 0 & 0 & 0 & 0 & 0 \\ 0 & 0 & 0 & 0 & 0 & 0 & 0 & 0 & \sqrt{\kappa_{s,\text{in}}} & 0 & \sqrt{\kappa_{s,\text{ex}}} & 0 & 0 & 0 \\ 0 & 0 & 0 & 0 & 0 & 0 & 0 & 0 & 0 & \sqrt{\kappa_{s,\text{in}}} & 0 & \sqrt{\kappa_{s,\text{ex}}} & 0 & 0 \\ 0 & 0 & 0 & 0 & 0 & 0 & 0 & 0 & 0 & 0 & 0 & 0 & \sqrt{\kappa_r} & 0 \\ 0 & 0 & 0 & 0 & 0 & 0 & 0 & 0 & 0 & 0 & 0 & 0 & 0 & \sqrt{\kappa_r} \end{bmatrix}, \tag{8}$$

and $\boldsymbol{A}(t) = [\delta\hat{a}_{o,\text{in}}, \delta\hat{a}_{o,\text{in}}^\dagger, \delta\hat{a}_{o,\text{ex}}, \delta\hat{a}_{o,\text{ex}}^\dagger, \delta\hat{a}_{e,\text{in}}, \delta\hat{a}_{e,\text{in}}^\dagger, \delta\hat{a}_{e,\text{ex}}, \delta\hat{a}_{e,\text{ex}}^\dagger, \delta\hat{a}_{s,\text{in}}, \delta\hat{a}_{s,\text{in}}^\dagger, \delta\hat{a}_{s,\text{ex}}, \delta\hat{a}_{s,\text{ex}}^\dagger, \delta\hat{a}_r, \delta\hat{a}_r^\dagger]$. Here, $g = g_0\sqrt{\bar{n}_p}$ is the parametrically enhanced electro-optic coupling strength and $\bar{n}_p$ the intra-cavity optical pump photon number. Equation 6 is solved in the Fourier domain, yielding

$$\boldsymbol{v}(\omega) = \boldsymbol{S}(\omega)\boldsymbol{A}'(\omega), \tag{9}$$

where $\boldsymbol{S} = [-\boldsymbol{M} - i\omega\mathbb{1}]^{-1}$ and $\boldsymbol{A}'(\omega) = \boldsymbol{K}\boldsymbol{A}(\omega)$. The output field can be obtained via the input-output theorem [6, 7],

$$\delta\hat{a}_{j,\text{out}}(\omega) = -\delta\hat{a}_{j,\text{in}} + \sqrt{\kappa_{j,\text{ex}}}\delta\hat{a}_j, \tag{10}$$

with j=o,s. The total conversion efficiency on resonance is calculated from a single matrix element as

$$\eta_{\text{tot}} = |S_{oe}|^2 = |S_{eo}|^2 = \Lambda^2\eta_e\eta_o \frac{4C\left(1 + C_J^{-1}\right)^2}{\left(1 + C + C_J^{-1}\right)^2}, \tag{11}$$

where $\eta_j = \kappa_{j,\text{ex}}/\kappa_j$ is the mode coupling efficiency, C is the multi-photon cooperativity defined as $C = 4\bar{n}_p g_0^2/(\kappa_o\kappa_e)$ and C_J, similarly, is the cooperativity related to the coupling J defined as $C_J = 4J^2/(\kappa_o\kappa_r)$. The factor Λ^2 is introduced by rewriting the external optical linewidth $\kappa_{o,\text{ex}} \to \Lambda^2\kappa_{o,\text{ex}}$.

Note, for high C_J, the avoided crossing in the lower FSR optical mode is fully split, resulting in perfect suppression and no participation of the lower frequency sideband mode. Hence, in this limit, the above formula for conversion efficiency reduces to the usual two mode model, $\eta_{\text{2-mode}} = 4\Lambda^2\eta_e\eta_o C/(1 + C)^2$[8]. Furthermore, for $C \ll 1$, we get back the linear dependence of conversion efficiency on C and no dependence on the value of C_J. The opposite limit is $C_J = 0$ which means there is no avoided crossing in lower FSR optical mode and, thus, equal participation of both optical modes on either side of optical pump. In this limit, we get the maximum possible gain and the conversion efficiency formula reduces to $\eta_{\text{J=0}} = 4\Lambda^2\eta_e\eta_o C$. In contrast to the case of just two modes, here the conversion efficiency does not saturate as C approaches 1.

E. Noise analysis

The noise spectrum of the output field can be obtained as $S_{jj,\text{out}}(\Omega) = \int_{-\infty}^{+\infty} \left\langle \delta\hat{a}_{j,\text{out}}^\dagger(t)\delta\hat{a}_{j,\text{out}}(t')\right\rangle e^{i\Omega t}dt$, via the Wiener-Khinchin theorem. The full expression of $S_{jj,\text{out}}(\Omega)$ is too long to show here. This is due to the complex mode coupling scheme and because the optical and microwave cavity linewidths are comparable in magnitude. The

output photon flux is obtained by integrating the output noise spectrum over the specific measurement bandwidth

$$N^j_{\text{out}} = \int_{-\infty}^{+\infty} \chi(\Omega) S_{jj,\text{out}}(\Omega) d\Omega, \tag{12}$$

where $\chi(\Omega)$ is the measurement filter function. In our experiment, we use a Gaussian filter with full width half max (FWHM) of 10 MHz. The equivalent input noise is calculated accordingly

$$N^j_{\text{in}} = N^j_{\text{out}}/\eta_{\text{tot}}. \tag{13}$$

The predicted output noise takes gain into account and we use the total conversion efficiency η_{tot} that also includes finite gain and coupling losses in order to infer the equivalent added noise referenced to the input port where the (quantum) signal is fed into the transducer.

In the absence of the optical pump (C=0), the output microwave noise spectrum is simply given as [8],

$$N^e_{\text{out},C=0}(\Omega) = \frac{4\kappa_{e,\text{in}}\kappa_{e,\text{ex}}}{\kappa_e^2 + 4\Omega^2}(N_b - N_{wg}) + N_{wg}. \tag{14}$$

We use the output noise in absence of the optical pump to infer the bath temperature and subsequently calculate the equivalent mode occupancy of the microwave mode

$$N_e = \frac{N_b\kappa_{e,\text{in}} + N_{wg}\kappa_{e,\text{ex}}}{\kappa_{e,\text{in}} + \kappa_{e,\text{ex}}}. \tag{15}$$

The calibration of the added noise during conversion critically depends on the measurement apparatus, which is discussed in detail in following sections.

Supplementary Note 2. EXPERIMENTAL SETUP

The experimental setups for low and high cooperativity conversion were slightly different and optimized for each measurement. The setup for low (high) cooperativity measurements is shown and described in Supplementary Fig. 2 (Supplementary Fig. 3). The main difference between the setups is whether the optics signal and the optics LO are separated. In case of the low cooperativity setup, we keep them always in the same optical fiber for better phase stability. This is possible because for lower cooperativities the optical pump powers are low enough such that they do not pose any threat to damage the photodetector (HSPD). In case of the high cooperativity setup we prepare the high power optical pump separately before combining it with the optical signal. Later, the optical signal is separated from the optical pump using an optical filter to prevent any damage to the photodetector (BPD). However, this reduces the phase stability timescales from few minutes to 10's of milliseconds. In spite of the reduced phase stability timescales we successfully demonstrate phase coherence between the input and converted signal at higher cooperativities. This is possible because the converted signals are much larger in magnitude which reduces the required averaging times considerably.

Supplementary Note 3. SYSTEM CHARACTERIZATION

Our experiment is divided in two broad parts - measurements at low cooperativity ($< 10^{-3}$) and measurements at high cooperativity (~ 1). We use a different set of optical modes for these experiments because of a large time gap of a few months between the experiments. In the following subsections, we detail the system characterization done for both parts of the experiment. Since we use the same device for this experiment, the electro-optic coupling rate g_0 is the same as reported in our previous manuscript [8].

A. Low cooperativity characterization

Before doing conversion measurements, we independently characterize the optical and microwave modes. We characterize these modes in both the time and frequency domain. Characterization of the optics mode in frequency domain is described in detail in reference [8]. We move the coupling prism via a piezo positioner, to change the

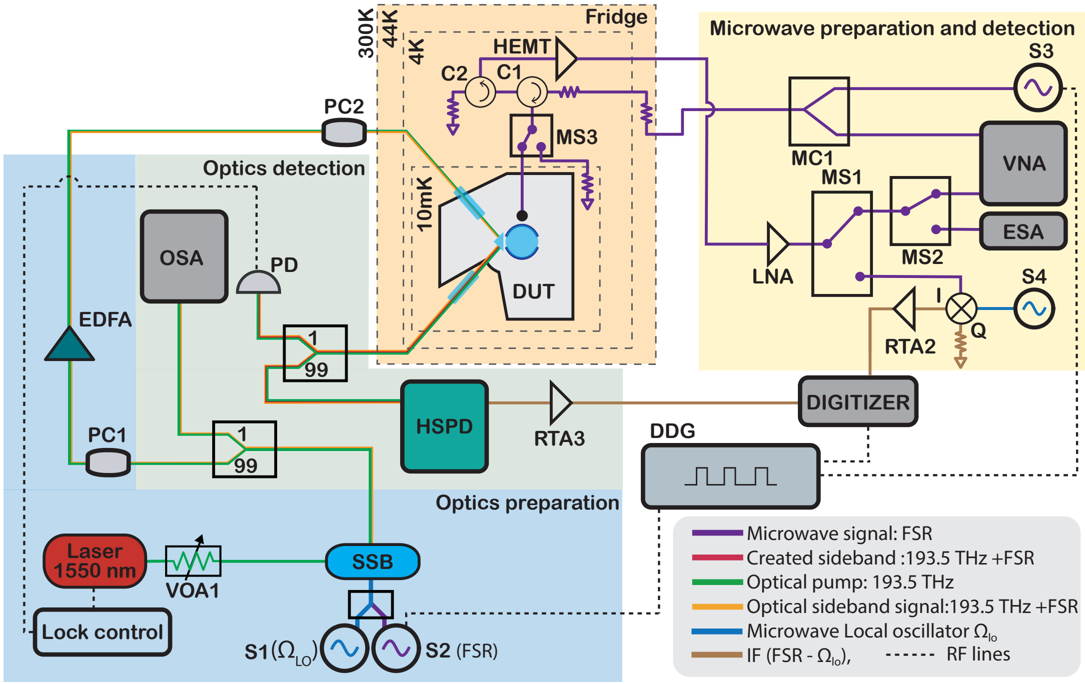

Supplementary Fig. 2. **Experimental setup for low cooperativity measurements.** A tunable laser at frequency ω_p is sent through a variable optical attenuator (VOA1) to control the power output. Thereafter, the laser is sent to a single sideband (SSB) modulator. The modulator operates in a mode where the central pump frequency is allowed to pass through and only the lower sideband is suppressed. The SSB is connected to two microwave sources. The arrangement allows us to independently control the optical signal tone and the optical local oscillator (LO) tone, 200 MHz detuned, independently. The optical signal source S2 is also connected to a digital delay generator (DDG) to make pulses at the right time. The modulated output from the SSB is divided in two parts - 1% is used to monitor the suppression ratio of the sidebands using an optical spectrum analyzer (OSA), 99% is sent to an Erbium-doped fiber amplifier (EDFA) and amplified before being sent to the dilution refrigerator (DR). In the DR, the light is focused via a gradient-index (GRIN) lens on the surface of the prism and coupled to the optical whispering gallery mode resonator (WGMR) via evanescent coupling. Polarization controllers PC1 and PC2 are adjusted to efficiently couple to the TE modes of optical WGMR. The output light from the optical WGMR is sent in a similar fashion into the collection grin lens. Outside the DR, the output light is separated in two parts - 1% is detected directly on a photo-diode (PD) to lock the laser to the optical pump mode and 99% is sent to a high speed photo-diode HSPD (400 MHz). The presence of the optical LO and signal in the same fiber means that the optical signal can be easily detected at the set frequency via downconversion. The output signal from HSPD is sent to an amplifier RTA3 before sent for digitization. On the microwave side, the signal is sent from the microwave source S3 which is also connected to DDG for accurately timed pulse generation (or from the VNA for microwave mode spectroscopy) to the fridge input line via the microwave combiner (MC1). The input line is attenuated with attenuators distributed between 3 K and 10 mK with a total of 60 dB in order to suppress room temperature microwave noise. Circulator C1 redirects the reflected tone from the cavity to the amplified output line, while C2 redirects noise coming in from the output line to a matched 50 Ω termination. The output line is amplified at 3 K by a HEMT-amplifier and then at room temperature again with a low noise amplifier (LNA). The output line is connected to switch MS1 and MS2, to select between an ESA, a VNA or a digitizer measurement via manual downconversion using MW LO S4 (200 MHz detuned). Lastly, microwave switch MS3 allows to swap the device under test (DUT) for a temperature $T_{50\,\Omega}$ controllable load, which serves as a broad band noise source in order to calibrate the output line's total gain and added noise [8].

external coupling rate $\kappa_{o,\mathrm{ex}}$, and fit the mode profile in frequency domain to extract the linewidth. The normalized mode spectrum is given as

$$\frac{\left|S_{oo}\left(\omega-\omega_o\right)\right|^2}{\left|\ \mathrm{S}_{oo}(\Delta\omega)\right|^2}=1-\frac{4\kappa_{o,\mathrm{ex}}\Lambda^2\left(\kappa_o-\Lambda^2\kappa_{o,\mathrm{ex}}\right)}{\kappa_o^2+4\left(\omega-\omega_o\right)^2}, \tag{16}$$

where ω_o is the optical mode resonance frequency. Fitting the measured optical spectrum, we determine the internal linewidth, coupling efficiency and mode matching factor, Λ, for the optical mode. Similarly, we also fit the microwave mode spectrum measured via a Vector Network Analyzer (VNA) to determine the total linewidth of microwave mode and its coupling efficiency. We then use the parameters obtained from frequency domain characterization to verify

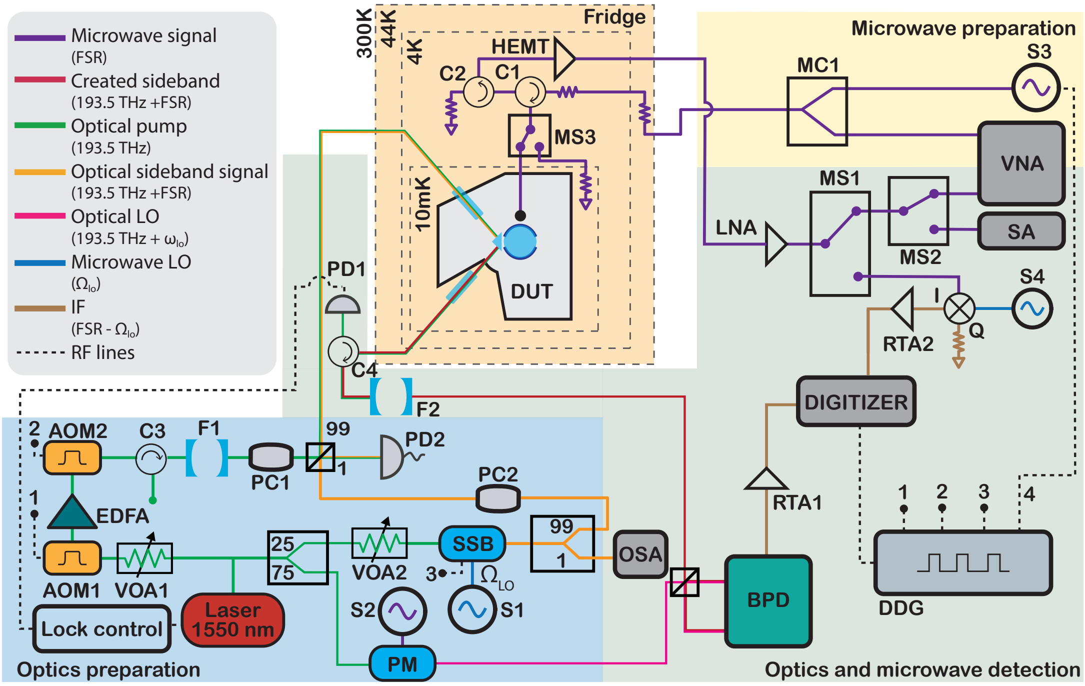

Supplementary Fig. 3. **Experimental setup for high cooperativity measurement.** A tunable laser at frequency ω_p is divided into two equal parts - one to serve as the optical pump and the other to produce the optical signal and the optical local oscillator (LO). The optical pump side (left) first passes through a variable optical attenuator (VOA1) to control the power sent in this arm and is then sent to an acousto-optic modulator (AOM1). The AOM is accurately pulsed using a digital delay generator (DDG). The produced optical pulsed are sent to an Erbium-doped fiber amplifier (EDFA) where they are amplified. The output of the EDFA is sent to another AOM2. The second AOM is also connected to DDG and acts as a window filter in time to suppress the broad band spontaneous emission noise from the EDFA. The optical pump pulse is further cleaned with a filter cavity F1 (50 MHz linewidth with ∼15 GHz FSR), which is locked to the laser frequency (circulator C3 ensures that reflected noise from cavity F1 is dissipated) before being combined with the signal arm and sent to the dilution refrigerator (DR). The signal arm (right of the laser) is first divided in two parts - the optical LO and the optical signal. 25% of the light in signal arm is used to produce the optical signal. After passing through attenuator VOA2, the optical signal is produced using a single sideband modulator (SSB). This time we suppress both the central pump frequency and the lower sideband frequency keeping only the upper sideband as the optical signal. The SSB is driven by a microwave source S1 which is also connected to the DDG to accurately pulse the optical signal. The optical signal is divided in two parts - 1% is reserved to monitor the sideband suppression ratio via an optical spectrum analyzer (OSA), 99% is sent to DR after combining with the optical pump. The 75% light on the right side of the laser is used to produce the optical LO via a phase modulator (PM). The PM is operated via a microwave source S2 with a power such that the central tone is suppressed. Finally, the optical LO is sent directly to the optical heterodyne setup. The optics inside the DR has been explained in the caption of Supplementary Fig. 2. The output light from the DR is sent to filter F2 (50 MHz linewidth with ∼40 dB supression) to reject the strong optical pump. The reflected optical pump from cavity F2 is captured by photodiode PD1 via the circulator C4. The reflected optical pump measurement is used to lock the laser to the optical pump mode. The cleaned optical signal is sent to the heterodyne setup and measured with a balanced photo-detector (BPD). The output signal is amplified via a room temperature amplifier RTA1 before being sent to a digitizer. The microwave side of the setup is explained in the caption of Supplementary Fig. 2.

our time-domain characterization described next.

Supplementary Fig. 4 shows the time domain characterization of the optics and microwave modes. We characterize the system by sending a square pulse of a coherent tone to the cavity on and off resonance. The off resonant pulse reflects without any modifications and, thus, is used as a measurement of the input pulse shape. We use Eqs. 5b and 5d as well as the input pulse shape to solve for modes $\bar{a}_o$ and $\bar{a}_e$ and, subsequently, calculate $\bar{a}_{o,\text{out}}$ and $\bar{a}_{e,\text{out}}$ as detailed in section Supplementary Note 1 to predict the pulse reflection on resonance and, finally, calculate the power reflected on resonance, $|\bar{a}_{j,\text{out}}|^2$. This prediction is used to fit the reflected power in Supplementary Fig. 4. The fitted system parameters are shown in Supplementary Tab. 1. The parameters fully agree with those determined by frequency domain characterization except for the optical mode matching factor, Λ, which was determined to be 0.806 from the frequency domain characterization and 0.838 from the time domain characterization. We use the value obtained from time domain characterization which we believe is more accurate and directly applicable to the time domain conversion measurements.

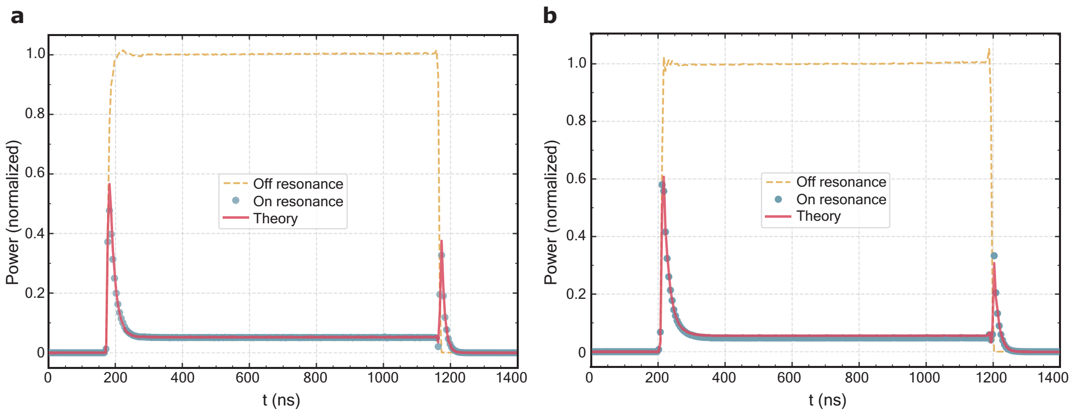

Supplementary Fig. 4. **Time domain system characterization for low cooperativity measurements. a** (**b**), Normalized pulse reflection of a square pulse from the optical (microwave) cavity on and off resonance. The red curve is a theoretical fit obtained using time domain input-output theory.

Supplementary Tab. 1. System parameters for low cooperativity measurements.

Parameter	Description	Value
$\omega_o/2\pi$	Optical signal frequency	193 THz
$\kappa_o/2\pi$	Optical signal linewidth	15.55 MHz
η_o	Optical coupling efficiency	0.55
Λ	Optical mode mismatch factor	0.838
$\omega_e/2\pi$	Microwave mode frequency	8.803 GHz
$\kappa_e/2\pi$	Microwave signal linewidth	12.12 MHz
η_e	Microwave coupling efficiency	0.369
$g_0/2\pi$	Electro-optic coupling rate	37 Hz

The shape of reflected pulse on resonance in Supplementary Fig. 4 is interesting to understand in more detail. The first peak occurs due to the rapid rise time of the input pulse which has much higher bandwidth than the cavity. Thus, all of it gets reflected before the cavity has the chance to absorb part of the input tone. The initial rise is interrupted as the cavity gets the time to absorb the input light and start re-emitting that light to cancel the input pulse reflection. This continues until a steady state is reached. At the end of the pulse the pulse power drops much faster than the cavity bandwidth. As a result, photon emission from the cavity does not get time to change. As the input pulse drops, there is a moment when the cavity emission perfectly cancels out the input reflection leading to a point of zero reflection (more clearly seen for shorter pulses, see Supplementary Fig. 6). As the input pulse drops further, the emission from the cavity takes over and the reflected power rises again. Finally, only the emission from the cavity is left which slowly decays with cavity linewidth.

For low cooperativities, we do not characterize the $\hat{a}_s$ mode hybridization since the conversion efficiency is approximately reduced to just the simple 3-mode beam-splitter like interaction where the TM coupling is irrelevant (see Eq. 11).

B. Low cooperativity conversion

Figure 5(a) and (b) shows the calibrated time dependent measurement of a converted signal pulse in case of microwave-to-optics and optics-to-microwave conversion, respectively. These pulses are measured with a bandwidth of 200 MHz and shown together with a fit to the numerical model (red line) that takes the measured input pulses (yellow dashed lines) with a rise time of 15 ns and 5 ns respectively as input data along with system characteristics from Table 1. We find excellent agreement of all four time dependent scattering parameters using only one fit parameter, the input optical loss. The 10% to 90% rise time of the converted pulses in both directions of 85 ns is limited by the linewidths of the optical and microwave modes in this case of comparably low cooperativity $C = 3.4 \times 10^{-4}$. Moreover, we also explicitly verify the faithful phase control and stability over subsequent pulses in both directions as shown in the insets.

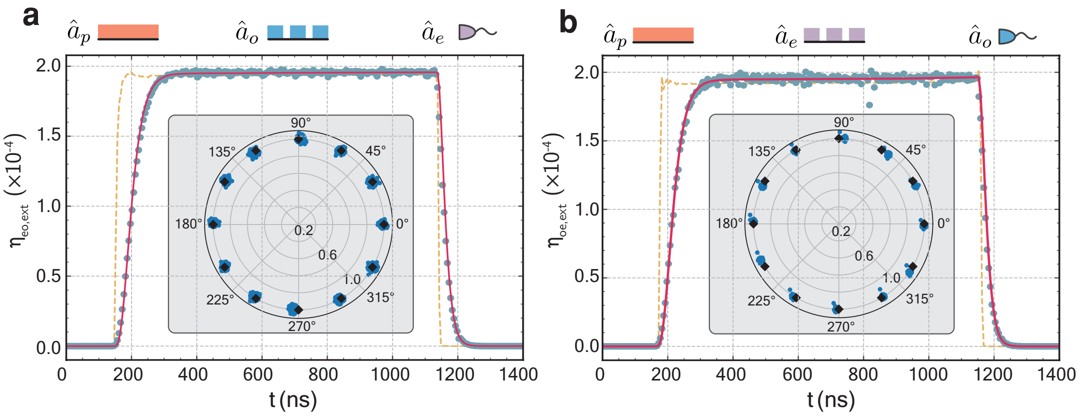

Supplementary Fig. 5. **Bidirectional conversion measurements for low cooperativity a** (**b**), Optics-to-microwave (microwave-to-optics) conversion for a continuous wave optical pump of $P_p = 134\,\mu$W. The measured input signal pulses are shown with dashed yellow lines and the converted signals with blue dots (red line is theory). Phase coherence and stability of the signal phase is shown in the insets. Rhombuses represent the phase imprinted on the input signal and the blue points represent phase values extracted from subsequently measured converted pulses.

C. High cooperativity characterization

Supplementary Fig. 6(a) and (b) shows the time domain characterization of the optical and microwave modes valid for high cooperativity measurements. The resulting system parameters obtained are shown in Supplementary Tab. 2. Supplementary Fig. 6(c) shows the optical Stokes mode $\hat{a}_s$ in the frequency domain. The mode is coupled to a degenerate TM optical mode $\hat{a}_r$, which results in an avoided crossing of the TE mode. We determine the strength of coupling between these modes, the total linewidth of the TM optical mode and its detuning by fitting the measured spectrum in the frequency domain.

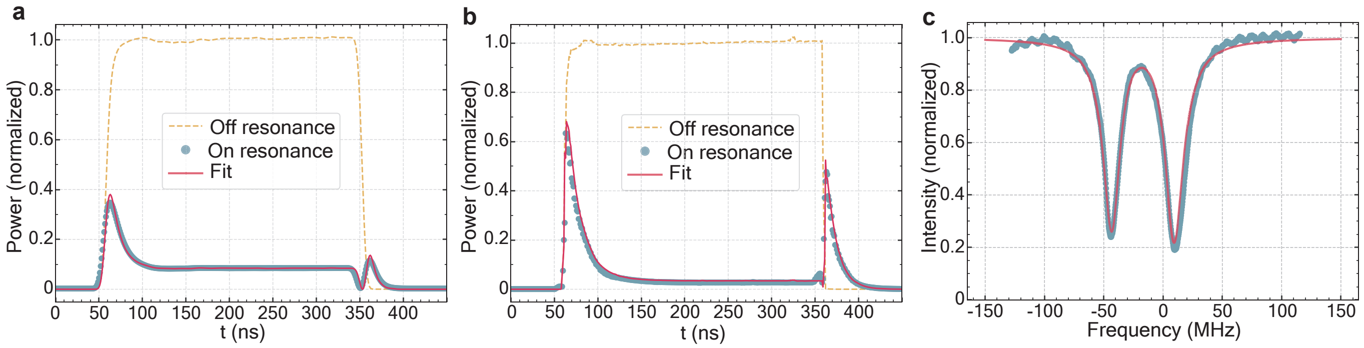

Supplementary Fig. 6. **System characterization for high cooperativity measurements. a** (**b**), Normalized pulse reflection of a square pulse from the optical (microwave) cavity on and off resonance. The red curve is a theoretical fit obtained using time domain input-output theory. **c**, Measured reflection spectrum around the red detuned TE optical mode $\hat{a}_s$. The mode shows an avoided crossing that is slightly detuned from the FSR at zero frequency because of its coupling to the near degenerate TM optical mode $\hat{a}_r$. The red line shows a theoretical fit of the split mode using Eq. 18.

The frequency response of the split mode is derived by considering two coupled modes where only one of them is

Supplementary Tab. 2. System parameters for high cooperativity.

Parameter	Description	Value
$\omega_o/2\pi$	Optical signal frequency	193 THz
$\kappa_o/2\pi$	Optical signal linewidth	25.8 MHz
η_o	Optical coupling efficiency	0.58
Λ	Optical mode mismatch factor	0.78
$\omega_e/2\pi$	Microwave mode frequency	8.795 GHz
$\kappa_e/2\pi$	Microwave signal linewidth	13.706 MHz
η_e	Microwave coupling efficiency	0.408
$g_0/2\pi$	Electro-optic coupling rate	37 Hz
$J/2\pi$	TE-TM optical mode coupling rate	26.21 MHz
$\kappa_r/2\pi$	Optical TM mode linewidth	9.96 MHz
$\Delta_s/2\pi$	Optical TE mode detuning	15.5 MHz
$\Delta_r/2\pi$	Optical TM mode detuning	19.5 MHz

pumped

$$\dot{\bar{a}}_s = -i\Delta_s\bar{a}_s - \tfrac{\kappa_o}{2}\bar{a}_s - iJ\bar{a}_r + \Lambda\sqrt{\kappa_{o,\mathrm{ex}}}\bar{F}_s, \tag{17a}$$

$$\dot{\bar{a}}_r = -i\Delta_r\bar{a}_r - \tfrac{\kappa_r}{2}\bar{a}_r - iJ\bar{a}_s. \tag{17b}$$

Solving the above set of equations in frequency domain, we find

$$a_s(\omega) = \frac{\Lambda\sqrt{\kappa_{o,\mathrm{ex}}}\hat{F}_s}{(i\Delta_s + \kappa_s/2) + \frac{J^2}{i\Delta_r + \kappa_r/2}}, \tag{18}$$

where $\Delta_j = \omega_j - \omega$. Subsequently, we calculate the output field as $\bar{a}_{s,\mathrm{out}}(\omega) = \Lambda\sqrt{\kappa_{o,\mathrm{ex}}}\bar{a}_s - \bar{F}_s$. We use this formalism to fit the split mode in the frequency domain. The resulting parameters are reported in Supplementary Tab. 2.

Supplementary Note 4. 4-PORT CALIBRATION

The total conversion efficiency is determined from a four-port calibration procedure of the transducer [8, 9]. For this purpose, the transducer is treated as a device with four ports - optics input (prism surface), optics output (prism surface), microwave input (coaxial coupler) and microwave output (same coaxial coupler) with optical (microwave) input transmission coefficient β_1 (β_3) of the setup as well as optical (microwave) output transmission coefficient β_2 (β_4) of the setup. Then, by measuring the optical and microwave transmission along with the conversion efficiency in both direction, the total conversion efficiency is calculated as

$$\eta_{\mathrm{tot}} = \sqrt{\frac{|S_{eo}(\omega_e)|^2 \cdot |S_{oe}(\omega_o)|^2}{|S_{oo}(\omega_{\Delta,o})|^2 \cdot |S_{ee}(\omega_{\Delta,e})|^2}}. \tag{19}$$

Here, the conversion efficiencies $|S_{eo}|^2$ and $|S_{oe}|^2$ are measured on resonance, while the transmission $|S_{oo}|^2$ and $|S_{ee}|^2$ are measured off resonance ($\omega_{\Delta,o} \gg \kappa_o$, $\omega_{\Delta,e} \gg \kappa_e$). Knowing any one of the four gain/loss terms (β_i) in the transmission lines, we can calculate all the other β_i. In the present case we use the microwave gain β_4 as the known quantity, which was measured independently (see appendix of Ref. [8]) and calculate the remaining β_i. We determine the following values: the optical input loss $\beta_1 = -6.33$ dB, the output optical gain $\beta_2 = 18.63$ dB, the microwave input loss $\beta_3 = -74.92$ dB and the microwave output gain $\beta_4 = 81.75$ dB.

Supplementary Note 5. TIME DEPENDENCE OF TRANSDUCTION EFFICIENCY

In the manuscript, we have shown conversion for two cases - when the optical pump is pulsed and the signal is continuous wave and when the signal is pulsed while the optical pump pulse is on. In the first case, when the signal is continuous wave, the resonator cavity is pre-loaded with the signal photons. This means that the resonator is in steady state with its characteristic internal and external losses. When the optical pump pulse arrives, the effective

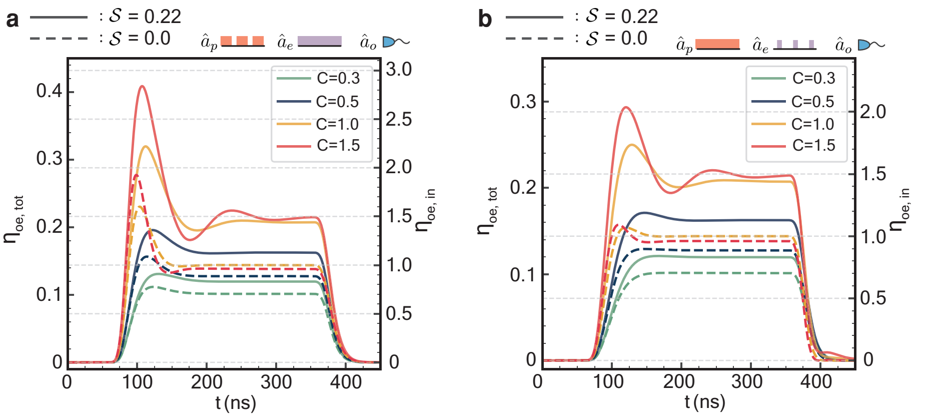

Supplementary Fig. 7. **Theoretical microwave to optics conversion** Left (right) axis shows the total transduction efficiency (internal transduction coefficient). The solid lines show conversion with a finite amount of gain with $\mathcal{S} = 0.22$ and the dashed lines show conversion without any gain with $\mathcal{S} = 0.0$. **a,** Case when optical pump is pulsed but microwave signal is continous wave and **b,** Case when optical pump is continous wave but microwave signal is pulsed instead. The steady state conversion without gain (dashed lines) always satures at unity for $C = 1$. The overshoots in the beginning of the pulse are higher for the case when the optical pump is pulsed.

internal loss of the cavity changes because a new loss/conversion channel opens up and a new steady state has to be reached. In the process of arriving to this steady state, temporarily, the conversion efficiency increases beyond the steady state as shown in Fig. 7(a). From another perspective, during this momentary overshoot, the coupling losses of the pre-loaded cavity are circumvented and the effective input coupling ratio becomes 1 temporarily.

For the case when the signal is pulsed while the optical pump is already on, the signal is never pre-loaded into the resonator. However, we still see an overshoot in Fig. 7(b) where the signal is pulsed while the optical pump is continuous wave. The reason for this overshoot is the onset of coherent oscillation between microwave and optics due to a high conversion rate. The oscillations are clearly seen at higher cooperativities $C \gtrsim 1$. Note that the first overshoot in case of Fig. 7(a) is always larger than in Fig. 7(b) for the same cooperativity because of the additional signal pre-loading effects.

Lastly, we identify the differences between the case with finite gain (solid lines) and the case with no gain/perfect suppression of the Stokes sideband (dashed line). For no additional gain in the transduction, the pure steady state efficiency saturates to unity as expected at $C = 1$ and starts to drop after that. However, for a system with finite gain, this saturation doesn't happen and the internal steady state transduction coefficient keeps increasing beyond unity.

Supplementary Note 6. OPTICAL HETERODYNE DETECTION

On the optical side, we detect the optical output signal using a balanced heterodyne setup, i.e. by beating the signal with a strong local oscillator with coherent optical field $\bar{a}_{\mathrm{LO}}$ at frequency of $\omega_o + \Omega_{\mathrm{LO}}$. This results in a balanced photocurrent $\delta I(t) = i(-e^{i\Omega_{\mathrm{LO}}t}\bar{a}^*_{\mathrm{LO}}\delta\hat{a}_{o,\mathrm{out}} + e^{-i\Omega_{\mathrm{LO}}t}\bar{a}_{\mathrm{LO}}\delta\hat{a}^\dagger_{o,\mathrm{out}})$. We thus obtain the symmetrized power spectral density $S_I(\Omega) = \frac{1}{2}\int_{-\infty}^{\infty}\langle\overline{\{\delta I\,(t+t')\,,\delta I\,(t')\}}\rangle e^{i\Omega t}dt$. The optical heterodyne efficiency or the equivalent noise floor level can be determined using the output gain β_2 and the absolute power measured in the baseline without any signal

$$P_{\mathrm{baseline}} = \hbar\omega_o\beta_2 BW\bar{n}_{\mathrm{add}}, \tag{20}$$

where BW the measurement bandwidth and $\bar{n}_{\mathrm{add}}$ the equivalent noise in the heterodyne baseline.

Using the output optical gain β_2, the equivalent noise floor in optics heterodyne $\bar{n}_{\mathrm{add}}$ is calculated to be 34.3 photons. The optical detection efficiency is low in our case for a number of reasons. There is a $\approx 3\,\mathrm{dB}$ loss while coupling the light from the device output, i.e. from the prism surface, to the optical fiber with a gradient index lens. An optical filter is used to reject the strong optical pump for the balanced heterodyne detection of the weak converted signals, which introduces another 4 dB of loss. We use the first order sideband generated from a phase modulator as the optical LO. Since the phase modulator produces many other optical tones, it reduces the optical balanced heterodyne efficiency to about 17 %. These are technical nonidealities of the setup that can be improved in the future.

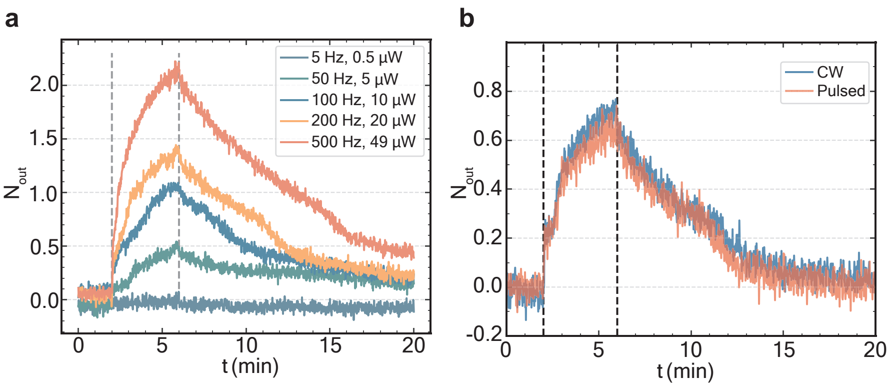

Supplementary Fig. 8. **Low-bandwidth microwave noise measurements.** The output noise of the microwave cavity is measured with a 100 kHz bandwidth. Each point is averaged for one second for better signal to noise ratio. **a**, Output microwave noise dynamics measured on long time scales as a function of pulse repetition rate for optical pump pulses corresponding to $C \sim 0.38$. The region between vertical dashed lines marks the time interval during which the optical pump pulses are turned on. **b**, Equivalence of added thermal microwave noise CW optical power and optical pulses with same average optical power.

Supplementary Note 7. MICROWAVE NOISE MEASUREMENTS

On the microwave side we perform phase-insensitive amplification of the weak microwave signal using a cryogenic low-noise high-electron mobility transistor amplifier [10]. The amplified microwave field is sent through subsequent amplifiers, and mixed with a microwave LO at room temperature. We calibrate the microwave output noise using total detected system noise 12.74 photons $s^{-1}Hz^{-1}$ which is the sum of the added noise due to microwave detection chain is 12.24 photons $s^{-1}Hz^{-1}$ and vaccum noise, as shown in the appendix of Ref. [8]. Finally, N_{out}^{eo} is calculated after subtracting this background noise to report only the added noise due to the transducer. In all of the noise measurements in the main text we report the measured added noise due to the transducer, i.e. with the constant added noise due to loss and amplifiers subtracted.

In the main text we show microwave noise measurements with two different bandwidths - 10 MHz for fast noise detection ($\sim$ 100 ns time resolution) and 100 kHz for slower noise detection but with better SNR. The fast 10 MHz measurements are triggered to capture the output noise response during the pulse. On the other hand, the 100 kHz measurements are run continously to measure the average thermal response of the triggered optical pump pulses. Due to their lower bandwidth and continous measurement nature, they provide a superior SNR performace to 10 MHz measurements and, thus, are used to determine the thermal noise occupancy for much lower average optical pump powers. The main source of error for the slow, low occupancy measurements is the systematic absolute error due to long term drifts of the noise baseline that is subtracted. We measure and average the noise baseline over multiple hours (like the measurements) on subsequent days to determine the absolute standard error of ± 0.02 photons $s^{-1}Hz^{-1}$. This error along with the statistical error of the actual noise measurement is propagated to get the final error bars. In the case of fast measurements, which were averaged only over a few tens of minutes, we take into account a larger observed variation of the noise baseline on that timescale of ± 0.1 to ± 0.2 photons $s^{-1}Hz^{-1}$.

The slow 100 kHz measurements allow us to study noise dynamics of the system on a long time scale. Supplementary Fig. 8(a) shows the average microwave noise output as the optical pump pulses corresponding to $C \sim 0.38$ with different repetition rates are turned on (marked region between vertical dashed lines). We observe that our system does not heat up immediately as soon as the pulses are turned on, rather it slowly reaches the steady state in a few minutes after the pulses are turned on. Moreover, the cooling time is even longer and it can take up to an hour to come back to the equilibrium occupancy depending on the average optical pump power applied. The specific time scales are expected to depend critically of the thermal contact and conductivity of the localized heat source (the dielectric-superconducting sample) to the cold bath (the mixing chamber plate), as well as on the cooling power of the dilution refrigerator [11].

Since this bulk electro-optic system has such long heating and cooling timescales, for short and fast repeating pulses, it is only the average optical power that determines the added thermal noise due to optical heating. Supplementary Fig. 8(b) shows the equivalence between heating via optical pulses (red) and continuous optical power (blue) during the time interval marked by the two vertical dashed lines. We observe the same dynamics of the output microwave noise if the power level of average optical power is matched. This experiment shows that thermal noise can be tuned by changing the pulse repetition rates while keeping the same level of cooperativity. This feature of the transducer allows to see amplified vacuum noise with negligible thermal noise by decreasing the pulse repetition rate and maintaining the high cooperativity.

For the highest repetition rates, the output microwave noise reaches close to 2 quanta. This corresponds to a local effective mode temperature on the order of 1 K. These temperatures compare to a much colder mixing chamber temperature of dilution refrigerator where the device is thermalized because of finite thermal conductivity of the dielectric resonator as well as the bulk superconducting aluminum cavity. We do, however, see a marginal increase in the mixing chamber temperatures as we increase the average optical pump power sent to the device. For a fastest repetition rate of 500 Hz, the mixing chamber temperature increased to at most 60 mK from its base temperature of 7 mK.

Supplementary Note 8. KERR EFFECT

In Fig. 2 of the main text we show conversion up to a cooperativity of 0.92. This is because after a certain threshold input power, which also depends on the optical pump pulse length, we observe an extreme amplification at the end of the conversion pulse. Supplementary Fig. 9 shows this effect for both microwave-to-optics and optics-to-microwave conversion cases. Since the amplification only happens at the end of the converted pulse, we are able to avoid it by applying a shorter optical pump pulse. At this point, the optical pump power could be further increased until the amplification appears again. The threshold power and hence the maximum achievable electro-optic cooperativity (without this amplification) therefore depends on the optical pump pulse length.

After a number of tests we came to the conclusion that this effect is most likely due to the third-order $\chi^{(3)}$ nonlinearity in lithium niobate, an effect that is commonly utilized in optical parametric amplifiers [12]. We verified that amplification in the optical signal is present even when there is no coherent signal drive present and for cases when the microwave mode is far detuned from the optical FSR. This proves that the effect is independent of the usual $\chi^{(2)}$ nonlinear interaction. However, when the microwave mode is matched with the optical FSR, we see amplification in the microwave signal as well due to strong optical sideband combined the $\chi^{(2)}$ mediated beam splitter interaction (conversion of the amplified optical signal). Note that the cooperativity threshold for amplification of $C \approx 1$, as shown in Supplementary Fig. 9, is a coincidence and only valid for 100 ns optical pump pulses. For longer optical pump pulses, the cooperativity threshold for amplification becomes smaller and well below unity.

This parameter regime of seeing effects of both $\chi^{(2)}$ and $\chi^{(3)}$ nonlinearities together is, to best of our knowledge, novel. Producing coherent phase-locked microwave and optical drives together requires a systematic investigation and may prove useful in future.

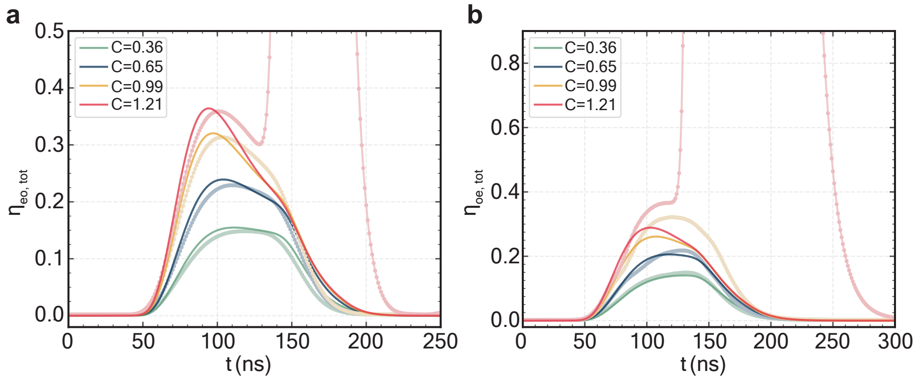

Supplementary Fig. 9. **Parametric optical amplification due to the $\chi^{(3)}$ non-linearity. a** (**b**), Time domain conversion in the microwave to optics (optics to microwave) direction for 100 ns optical pump pulses of different power and C. The points joined by light colored lines are measured experimentally. The thin bright lines are theoretical only taking into account the $\chi^{(2)}$ effect. After $C \approx 1$, we observe a delayed parametric amplification event. The measured transmission reaches as high as 11.4 in case of microwave to optics conversion and 13.1 in case of microwave to optics conversion (not visible).

REFERENCES

---

[1] Ilchenko, V. S., Savchenkov, A. A., Matsko, A. B. & Maleki, L. Whispering-gallery-mode electro-optic modulator and photonic microwave receiver. *J. Opt. Soc. Am. B, JOSAB* **20**, 333–342 (2003).

[2] Tsang, M. Cavity quantum electro-optics. *Phys. Rev. A* **81**, 063837 (2010).

[3] Rueda, A. *et al.* Efficient microwave to optical photon conversion: an electro-optical realization. *Optica* **3**, 597–604 (2016).

[4] Werner, C. S. *et al.* Control of mode anticrossings in whispering gallery microresonators. *Optics express* **26**, 762–771 (2018).

[5] Rueda Sanchez, A. R. *Resonant electrooptics.* PhD Thesis, Friedrich-Alexander-Universität Erlangen-Nürnberg (FAU) (2018).

[6] Gardiner, C. W. & Collett, M. J. Input and output in damped quantum systems: Quantum stochastic differential equations and the master equation. *Physical Review A* **31**, 3761 (1985).

[7] Tsang, M. Cavity quantum electro-optics. II. Input-output relations between traveling optical and microwave fields. *Phys. Rev. A* **84**, 043845 (2011).

[8] Hease, W. *et al.* Bidirectional electro-optic wavelength conversion in the quantum ground state. *PRX Quantum* **1**, 020315 (2020).

[9] Andrews, R. W. *et al.* Bidirectional and efficient conversion between microwave and optical light. *Nat. Phys.* **10**, 321–326 (2014).

[10] Clerk, A. A., Devoret, M. H., Girvin, S. M., Marquardt, F. & Schoelkopf, R. J. Introduction to quantum noise, measurement, and amplification. *Rev. Mod. Phys.* **82**, 1155–1208 (2010).

[11] Mobassem, S. *et al.* Thermal Noise in Electro-Optic Devices at Cryogenic Temperatures. *arXiv:2008.08764* (2020).

[12] Kippenberg, T. J., Spillane, S. M. & Vahala, K. J. Kerr-Nonlinearity Optical Parametric Oscillation in an Ultrahigh-Q Toroid Microcavity. *Physical Review Letters* **93**, 083904 (2004).
